# CD38-targeted attenuated interferon alpha immunocytokine activates both innate and adaptive immune cells to drive anti-tumor activity

**DOI:** 10.1371/journal.pone.0321622

**Published:** 2025-05-02

**Authors:** James F. Sampson, Hong Zhang, Dongmei Zhang, Mingying Bi, Adam Hinthorne, Sakeena Syed, Yuhong Zhang, Nibedita Chattopadhyay, Sabrina Collins, Sarah Pogue, Pia Björck, Michael Curley

**Affiliations:** 1 Oncology Drug Discovery Unit, Takeda Development Center Americas, Inc. (TDCA), Lexington, Massachusetts, United States of America; 2 Discovery R and D, Teva Pharmaceutical Industries, Ltd., Redwood City, United States of America; University of California Santa Barbara, UNITED STATES OF AMERICA

## Abstract

Recombinant interferon alpha (IFNα) has been used to treat cancer patients for over 30 years; however, its clinical utility has been limited by a narrow therapeutic index. Given the recognized anti-tumor and immunomodulatory impacts of IFNα, the development of novel strategies to harness these attributes while minimizing associated toxicity could provide significant benefit for patients. The concept of attenuating IFNα binding affinity for its receptor was conceived to address this challenge and led to the development of CD38-targeted Attenukine™, a CD38-targeted antibody attenuated IFNα immunocytokine. In this study, we sought to delineate the effects of targeting Attenukine^TM^ specifically to tumor cells and/or immune cells using an antibody to CD38, a cell surface glycoprotein expressed on certain tumor and immune cells, using different mouse models and anti-human or anti-mouse CD38-targeted Attenukine™. Our results demonstrate that an anti-human CD38 Attenukine^TM^ inhibits tumor growth through direct anti-proliferative effects of IFNα on CD38 + tumor cells as well as by indirectly modulating the anti-tumor immune response. In various *in vivo* models leveraging syngeneic mice bearing tumors with or without CD38 expression, administration of CD38-murine Attenukine^TM^ mediated anti-tumor efficacy with increased immune activation and intra-tumoral infiltration. These data point to a potential dual mechanism of action for CD38-targeted Attenukine™, involving both tumor- and immune-directed effects, and highlight the potential benefit of a CD38-targeted attenuated IFNα therapy to deliver the known effects of IFNαtreatment to a broad spectrum of patients, while limiting the toxicity typically associated with recombinant IFNα.

## Introduction

Interferon alpha (IFNα) is a pleiotropic cytokine that, through binding to the widely expressed IFNα receptor (IFNAR), mediates broad-spectrum signal activation with diverse outcomes including immune activation, direct tumor cell cytotoxicity, and inhibition of tumor cell proliferation [1]. Despite its potent anti-tumor and pro-inflammatory attributes and some promising signs of activity in certain tumor indications, the clinical utility of recombinant IFNα has been limited due to a narrow therapeutic index, driven by significant IFNα-related toxicities including nausea, severe flu-like symptoms, vasculopathic complications (e.g., thrombocytopenia and leukopenia), and negative impacts on the nervous system, resulting in depression and anxiety [2–5].

The role of type I IFNs, such as IFNα, on immune cells and tumor cells has been studied extensively. Type I IFNs stimulate the maturation and activation of natural killer (NK) cells directly and enhance NK cell priming through upregulation of membrane-bound interleukin (IL)-15 on dendritic cells (DCs) [6]. In addition to IL-15 upregulation, IFNα also enhances DC activation and cross-presentation of antigens to T cells [7]. Activated IFNAR signaling on T cells enhances their activation and cytotoxicity potential and can reduce regulatory T cell (Treg) suppressive capacity [8,9]. Although the response to IFNα treatment can vary across different cell lines and individual cells within a tumor, IFNα treatment has been shown to reduce the proliferation and/or promote the apoptosis of tumor cells [10]. Thus, IFNα-mediated tumor clearance may be realized through the convergence of both tumor-directed and immune-directed mechanisms.

In this study, we sought to discern the mechanistic impact of tumor-directed versus immune-directed effects of a CD38-targeted Attenukine^TM^, leveraging different immunocompromised and immunocompetent mouse models and various human or mouse CD38-directed Attenukine™. Our data indicate robust anti-tumor activity of CD38-directed Attenukine™ across multiple *in vivo* mouse models, several of which do not express CD38 on the tumor cells. Subsequent pharmacodynamic and immunodepletion studies underscore the innate and adaptive immune effects of a CD38-directed Attenukine™ in driving these anti-tumor responses.

Taken together, these data indicate that a CD38-directed Attenukine^TM^ may deliver robust anti-tumor activity by leveraging both tumor- and immune-directed effects of attenuated IFNα, without eliciting systemic IFNα-related toxicities.

## Materials and methods

### Generation of treatment agents

Anti-human CD38-human Attenukine^TM^ (hCD38-hAtt) was generated as previously described [11]. Briefly, reference anti-CD38 antibody variable regions were generated by polymerase chain reaction (PCR) from published V region sequences (WO 2013/059885). The human IFNα2b gene was isolated from HEK293 genomic DNA, and one attenuating point mutation was introduced by PCR at a residue which interacts with the high-affinity IFNα receptor chain, IFNAR2 [12]. Anti-CD38 and IFNα2b gene fragments were cloned into the pTT5 mammalian expression vector [13] containing human immunoglobulin (Ig)G4 and kappa Ig constant region genes. Selection of an IgG4 backbone was made to minimize functionality associated with Fc gamma receptor engagement [14]. Antibody- IFNα fusion proteins were transiently expressed in HEK293-6E cells [13] and purified using Protein G-Sepharose columns (GE Healthcare).

Generation of murine Attenukine^TM^ (mAtt) was achieved using PCR to introduce a V143A mutation into the murine IFNα6 sequence. Murine IFNα6 (hereafter referred to as mIFNα) was selected as it is the murine isoform of IFNα with the highest degree of sequence homology to human IFNα2b. Anti-human CD38-murine Attenukine^TM^ (hCD38-mAtt) was generated by cloning the equivalent anti-human CD38 antibody variable regions from hCD38-hAtt and the attenuated mIFNαA6 sequences into CHO K1 cells (WuXi Biologics) and purified using Protein G columns. Anti-murine CD38-murine Attenukine^TM^ (mCD38-mAtt) was generated by cloning the mAtt fused to an anti-mouse CD38-specific antibody (generated internally at Takeda in a hybridoma campaign) into Expi293 cells (WuXi Biologics). Recombinant mouse IFNα was purchased from BioLegend, and mouse IgG1 isotype control was purchased from Bio X Cell.

### Mice

Six- to 8-week-old female BALB/c (BALB/cAnNCrl), C57B6/J (C57B6NCrl), NCG (NOD-*Prkdc*^*em26 Cd52*^*Il2rg*^*em26 Cd22*^/NjuCrl), and C3H (C3H/HeNCrl) mice were purchased from Charles River Laboratories. All mice were housed under specific pathogen-free conditions in cages of up to four animals. Studies were approved by Takeda’s Institutional Animal Care and Use Committee (IACUC), and their guidelines on the ethical use and care of animals were followed.

Tumor cell inoculation was performed under isoflurane anesthesia. Tumor volumes and body weights were measured bi-weekly. Mice were euthanized at experimental endpoint (as defined in the *in vivo* tumor models section) by carbon dioxide inhalation followed by cervical dislocation. Animals that reached humane endpoint prior to experimental timepoint were euthanized and excluded from data analysis. Humane endpoint was defined as: tumors constituted more than 10% of body weight, exceeded 2 cm in any direction, or impaired basic functions such as eating, drinking, urination, defecation, or locomotion, weight loss exceeded 15% within a 24-hour period or 20% relative to baseline body weight at the onset of treatment.

### Cell lines

LP-1 and JJN-3 multiple myeloma cell lines were obtained from the German Collection of Microorganisms and Cell Cultures (DMSZ) and maintained in RPMI 1640 (Gibco, L-glutamine included) supplemented with 10% fetal bovine serum (FBS; Gibco), penicillin (100 U/ml), and streptomycin (100 mg/ml) (Gibco, 100 × pen/strep solution). CT26 (H-2^d^) colorectal carcinoma, A20 (H-2^d^) B lymphocyte sarcoma, B16F10 (H-2^b^) melanoma, and MM1.S multiple myeloma cell lines were obtained from the American Type Culture Collection and maintained in supplemented RPMI 1640. ANBL-6 multiple myeloma cells were purchased from Millipore Sigma. The MC38 (H-2^b^) colon adenocarcinoma cell line was obtained from the National Cancer Institute and maintained in supplemented RPMI 1640. 38C13-hCD38 cells were generated at Teva Pharmaceuticals by stably expressing human CD38 on the surface of 38C13 tumor cells [kindly provided by Professor Ronald Levy (Stanford University)], with surface expression confirmed by flow cytometry (S1 Fig).

### *In vivo* tumor models

All tumor cells were injected subcutaneously in 200 μl final volume with phosphate-buffered saline (PBS) into the right flank of mice. Unless otherwise stated, the following tumor cell numbers for the different mouse strains were used for inoculation: 5 x 10^6^ for ANBL-6 (NCG), LP-1 (NCG), and MM1.S (NCG); 5 x 10^5^ for JJN-3 (NCG); 1x10^3^ for 38C13-hCD38 (C3H); 3 × 10^5^ for CT26 (BALB/c), A20 (BALB/c), MC38 (C57BL/6), and B16F10 (C57BL/6). Ten to 15 mice per treatment group were used for anti-tumor activity studies and 7–10 mice per treatment group were used for pharmacodynamic studies. Tumor growth was monitored using electronic calipers and tumor volume was calculated using the formula: π/6 × (length × width^2^). When tumors reached an average volume of 50–100 mm^3^, mice were randomized into treatment groups to achieve comparable distribution of mean starting tumor volumes per condition. Treatment agents were injected intraperitoneally at indicated doses and schedules in a total volume of 200 μl. Where indicated, control agents were dosed at concentrations which were molar equivalent (either antibody or IFNα) to the dose of the CD38 Attenukine^TM^ administered, either murine or human. Mice assigned to vehicle groups were administered saline solution in 200 μl final volume. In anti-tumor activity studies, mice were euthanized when tumor volumes reached a pre-specified tumor volume of ≥ 2,000 mm^3^. A complete response (CR) to treatment was defined for mice in which the recorded tumor volume was below 60 mm^3^ and continued to regress until the end of the study.

### *In vivo* depletion of immune cell subsets

For studies evaluating the contribution of specific immune cells to mCD38-mAtt or hCD38-mAtt mediated anti-tumor activity, immune cell-specific depleting antibodies were used. CD8 T cells were depleted using 300 μg anti-CD8 mAb clone 53–6.7 (BioXCell [15]), CD4 T cells were depleted using 300 μg anti-CD4 clone GK1.5 (BioXCell [16]), and NK cells were depleted using 50 μl polyclonal anti-Asialo GM-1 (BioLegend [17]). Mice were administered respective depleting agents at the indicated doses 1 day prior to treatment initiation and weekly thereafter.

### Tumor dissociation

Tumors were dissociated using the mouse Tumor Dissociation Kit and gentleMACS system, according to manufacturer’s protocol (Miltenyi). GentleMACS C tubes containing minced tumors were incubated in digestion buffer using the heating function of the gentleMACS Octo Dissociator. Resulting single-cell suspensions were passed through 70 μM filters (Miltenyi) and evaluated further *ex vivo*, as described below.

### *Ex vivo* analysis of antigen-specific CD8 T cells in the CT26 tumor model

Tumors were collected at indicated time points after treatment and dissociated to single-cell suspensions, which were stained with either AH1 gp70423–431/H-2L^d^ dextramer (Immudex) or stimulated with AH1 peptide and intracellularly stained. Cells were stained with a 1:10 dilution of dextramer in PBS containing 5% FBS for 10 minutes at room temperature, washed twice, and stained for expression of extracellular markers in flow cytometry buffer at 4°C. For peptide stimulations, cells were incubated with 1 mM gp70423–431 AH1 peptide (New England Peptide) in complete RPMI containing brefeldin A for 5 hours, followed by staining for intracellular granzyme B. The percentage of granzyme B^+^ cells in cells stimulated with no peptide was subtracted from that of cells stimulated with AH1 to determine the amount of granzyme B produced by AH1-specific cells.

### Flow cytometry

Cells were stained with the following anti-mouse antibodies from BioLegend unless otherwise stated: CD4 (RM4–5), CD8 (KT15, ThermoFisher), Foxp3 (REA788, Miltenyi), CD25 (PC61), Granzyme B (GB11), Ki-67 (REA183, Miltenyi), CD11b (M1/70), CD11c (REA754, Miltenyi), CD49b (DX5), B220 (RA3-6B2), CD45 (30-F11), CD3 (145-2C11), Ter-119 (TER-119), NKp46 (9E2), TNF (MP6-XT22) and IFNGAMMA (XMG1.2). Single-cell suspensions were incubated with Fc Block (mouse 2.4G2) and live/dead Ghost Dye red710 (Tonbo Biosciences) in PBS for 10 minutes at 4°C, followed by staining for extracellular markers for 30 minutes at 4°C in FACS buffer. For intracellular staining including nuclear proteins, cells were fixed and permeabilized using the Foxp3 Staining Kit (BioLegend), according to the manufacturer’s protocol. When staining for intracellular cytosolic proteins only, cells were permeabilized using Fixation and Intracellular Staining Perm Buffer (BioLegend). Human tumor cell lines were stained with anti-CD38 (HIT2) and anti-IFNAR2 (122; ThermoFisher). Samples were evaluated on an LSR Fortessa flow cytometer (BD Biosciences) and data were analyzed using FlowJo analysis software (Tree Star) version 10.8.1. The gating strategy used to distinguish different immune cell subpopulations is provided in S2 Fig.

### Gene expression analysis

Tumors were evaluated for cytotoxic and suppressive molecule expression, relative to housekeeping gene, using the mouse I-O GeoMx Digital Spatial Profiling panel (Nanostring Technologies) according to the manufacturer’s protocol.

### Cell viability assay

Cell viability was measured using the CellTiter-Glo® kit (Promega), according to the manufacturer’s protocol. Luminescence was determined using a PHERAstar FS plate reader (BMG Lab Tech).

### Statistical analyses

Statistical analyses were performed using the GraphPad Prism 7.05 software (GraphPad Software, Inc.). For multiple comparison test, Kruskal-Wallis non-parametric test with Dunn’s multiple comparison post-test was used. Comparisons were made between control and treatment groups. For survival curves, groups were compared using the log rank (Mantel-Cox) test. Linear regression was used for correlation analysis. Percent tumor growth inhibition (%TGI) was calculated as:

(the median time to endpoint (TTE) for a treatment group minus the TTE for the control group) x 100

(TTE for the control group)
The median TTE in days was defined as:
(log_10_ (endpoint volume, mm ^3^ )-b)
m

Where b is the intercept and m is the slope of the line obtained by linear regression of log-transformed tumor growth data set. Statistical significance is indicated as *P < 0.05, **P < 0.01, ***P < 0.001, and ****P < 0.0001. Population statistics are displayed as mean and standard error mean or standard deviation, as indicated.

## Results

### hCD38-hAtt demonstrates anti-tumor activity in xenograft models of multiple myeloma

**Fig 1:**
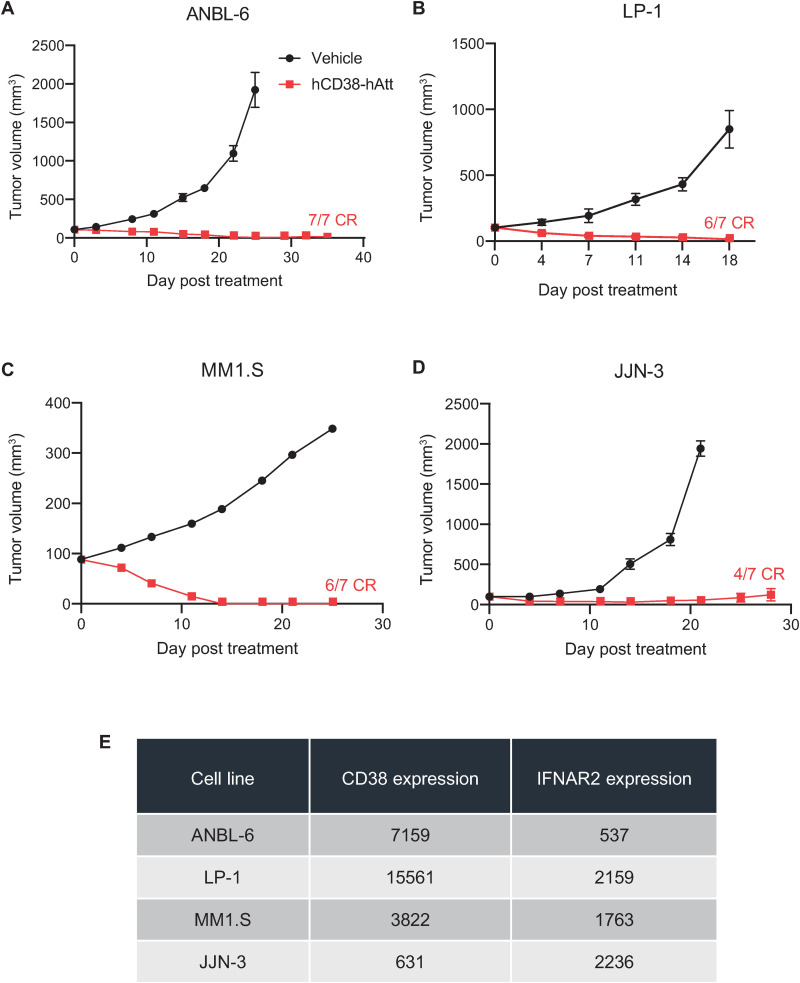
*In vivo* hCD38-hAtt treatment results in robust anti-tumor activity in xenograft tumor models of multiple myeloma. Immunocompromised NCG mice were inoculated with **A**, ANBL-6, **B**, LP-1, **C**, MM1.S, or **D**, JJN-3 tumor cells, at cell numbers outlined in the Materials and Methods section. When tumors reached an average volume of ~ 100 mm^3^ (day 0), mice were randomized into treatment groups and treated via intraperitoneal injection with vehicle or 10 mg/kg hCD38-hAtt twice weekly for up to six doses. Tumor volumes were measured twice weekly until humane endpoint was reached. **E,** Tabulated data summary of CD38 and IFNAR2 expression (mean fluorescence intensity) of each tumor cell line, as measured by flow cytometry.

Anti-human CD38-hAtt (hCD38-hAtt) has been shown to reduce the proliferation of leukemia and lymphoma cell lines *in vitro*, and to inhibit the growth of CD38-expressing xenograft tumors in immunodeficient mice [11]. Given the historical use of recombinant IFNα as a treatment option for patients with multiple myeloma, the impact of hCD38-hAtt on the growth of multiple myeloma xenograft tumors was evaluated. In studies evaluating the anti-tumor activity of hCD38-hAtt against ANBL-6, LP-1, MM1.S, and JJN-3 multiple myeloma tumors *in vivo*, CRs were observed in most hCD38-hAtt-treated mice (Fig 1 A–D). Anti-tumor activity of hCD38-hAtt in these xenograft tumor models did not directly correlate with the levels of expression of CD38 or IFNAR2 of these tumor cell lines *in vitro* (Fig 1E).

### NK cells, CD4, and CD8 T cells contribute to anti-tumor activity of hCD38-mAtt treatment

**Fig 2:**
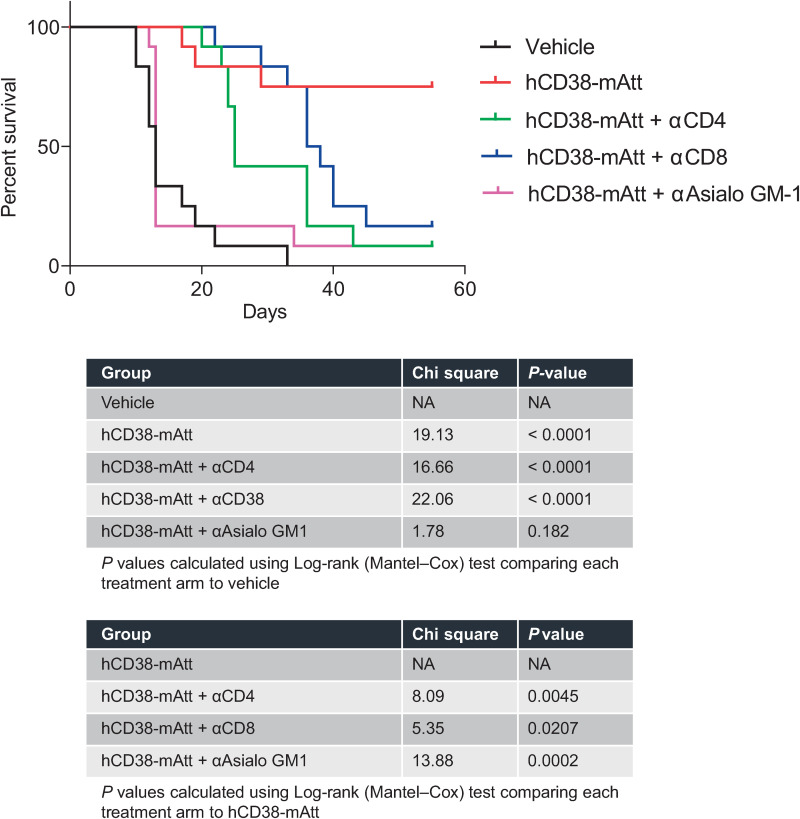
NK cells, CD4 T cells, and CD8 T cells contribute to the anti-tumor activity of hCD38-mAtt and in extending overall survival of treated mice in the 38C13-hCD38 tumor model. C3H (C3H/HeNCrl) mice bearing 38C13-hCD38 tumors were randomized into different treatment groups when tumors reached an average volume of ~ 100 mm^3^. One day prior to treatment (day -1), and every 7 days thereafter, mice were administered depleting αCD4, αCD8, or αAsialo GM-1 antibodies at doses shown to effectively and specifically deplete these cell populations [15–17]. On day 0, mice were administered 7 mg/kg hCD38-mAtt or PBS vehicle (equivalent final volume of 200 µ L) twice weekly by intraperitoneal injection for up to six doses. *P*-values were calculated using Log-rank (Mantel-Cox) test comparing each depletion treatment arm to vehicle or hCD38-mAtt, as indicated.

**Fig 3:**
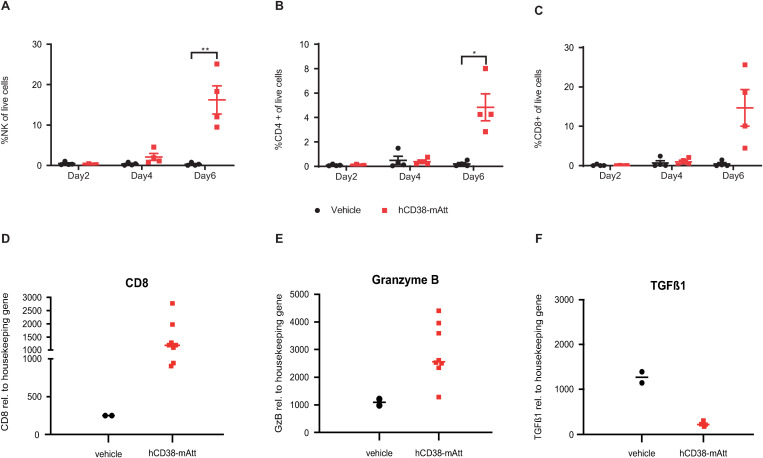
Increased intra-tumoral infiltration and activation of immune cells driven by hCD38-mAtt. C3H (C3H/HeNCrl) mice were inoculated with 38C13-hCD38 tumor cells and, when tumors reached an average volume of ~ 300 mm^3^, treated with vehicle (PBS, 200 µ L final volume) or treatment agent hCD38-mAtt on days 1 and 4. Tumors were collected at 2, 4, and 6 days post treatment initiation (day 0) from pre-specified groups of mice. The intra-tumoral numbers of **A,** NK cells, **B,** conventional CD4 T cells, and **C,** CD8 T cells as a percentage of live cells, was determined by flow cytometry. Gene expression of **D,** CD8α, **E,** granzyme B, and **F,** TGFβ1 was captured at day 6 post treatment in the tumor. Samples from each animal were run in triplicate. GzB, granzyme B.

To better distinguish the impact of tumor-directed versus immune-directed activity of a CD38-directed Attenukine^TM^, different immunocompetent animal strains and tumor models (expressing human or mouse CD38) were used in *in vivo* studies. In the first study leveraging the 38C13-hCD38 tumor model and evaluating hCD38-mAtt activity, hCD38-directed binding was only possible to hCD38 exclusively expressed on these engineered tumor cells. *In vitro*, 38C13-hCD38 cells demonstrated robust sensitivity to hCD38-mAtt and mIFNα alone (S3 Fig). *In vivo*, hCD38-mAtt treatment demonstrated a significant improvement in overall survival (OS) compared with vehicle control (Fig 2). This anti-tumor activity was partially abrogated by depletion of CD4 and CD8 T cells, and completely abrogated by depletion of NK cells, resulting in a decreased OS of treated mice (Fig 2). A second study leveraging this model evaluated the infiltration of immune cells into the tumor bulk as a potential consequence of hCD38-mAtt treatment. In these studies, mice bearing 38C13-hCD38 tumors were treated with vehicle or hCD38-mAtt. At 6 days post treatment, a significant increase in NK cell numbers within the tumors of hCD38-mAtt treated mice was noted compared with vehicle alone (Fig 3A). In addition, a significant increase in CD4 T cell numbers and a trend towards increased CD8 T cell numbers were demonstrated within tumors of hCD38-mAtt-treated mice compared with vehicle controls (Fig 3B–C). Finally, gene expression-based analyses of samples at 6 days post treatment pointed to an increase in CD8α (Fig 3D) and granzyme B (Fig 3E) expression in the tumors of hCD38-mAtt-treated mice compared with vehicle-treated mice. However, transforming growth factor (TGF) β1 was decreased when compared with vehicle-treated mice (Fig 3F).

### Administration of mCD38-mAtt results in modest to robust anti-tumor activity in syngeneic models with different intrinsic sensitivities to IFNα

**Fig 4:**
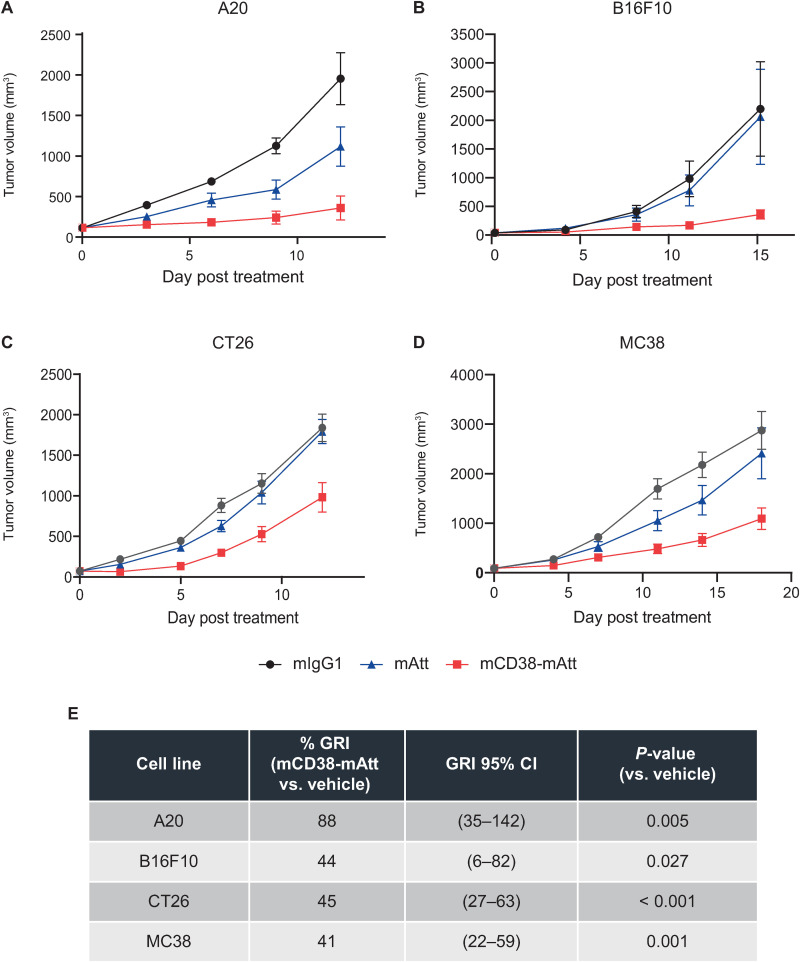
*In vivo* administration of mCD38-mAtt demonstrates differential anti-tumor activity across a range of syngeneic mouse tumor models. Different strains of immunocompetent mice (see Materials and Methods) were inoculated with **A**, A20, **B**, B16F10, **C**, CT26, or **D**, MC38 tumor cells, and when tumors reached an average volume of ~ 100 mm^3^, mice were randomized into treatment groups and treated with a single intraperitoneal dose of 8 mg/kg mIgG1 isotype control, 2 mg/kg mAtt alone (i.e., no antibody), or 10 mg/kg mCD38-mAtt. These dose levels were equilibrated based on dosing the molar equivalence of antibody (mIgG1 isotype to mCD38-mAtt) or mIFNα6 (mAtt alone to mCD38-mAtt). Tumor volume was measured until humane endpoint was reached in the vehicle-treated groups. **E,** Tabulated summary documenting percent GRI, and statistical significance thereof, following treatment with mCD38-mAtt versus vehicle control for each tumor model. CI, confidence interval; mIFNα6, murine interferon alpha 6; mIgG1, murine immunoglobulin G1.

Having demonstrated immune-directed impacts of treatment with hCD38-mAtt in a tumor model expressing a human CD38 target, additional *in vivo* studies were performed using a fully murine surrogate Attenukine^TM^, namely mCD38-mAtt. Syngeneic tumor models, which varied in their intrinsic sensitivity to murine IFNα stimulation (S4 Fig) but lacked human or murine CD38 surface expression, were treated with mCD38-mAtt to interrogate the immune-directed activity of a CD38-targeted Attenukine^TM^ in isolation. Initial *in vivo* studies in these models focused on the potential anti-tumor activity of a single intraperitoneal dose of mCD38-mAtt (10 mg/kg). In A20 tumor-bearing mice, two of eight treated mice had a CR (i.e., no visible tumor measurable) following administration of mCD38-mAtt (Fig 4A). The A20 cell line was determined to be the most sensitive to the anti-proliferative effects of murine IFNα treatment *in vitro* (S4A Fig), despite its lack of CD38 expression [18]. Robust to more modest improvement in *in vivo* activity, which significantly differed from vehicle-treated groups, was demonstrated following treatment of B16F10, CT26, and MC38 solid tumors (Fig 4B–D, respectively), which did not correlate with the comparative *in vitro* sensitivity of these cell lines to murine IFNα treatment (Fig 4E; S4B–D Fig).

### T cells and NK cells are important mediators of anti-tumor immunity in mCD38-mAtt-treated mice

**Fig 5:**
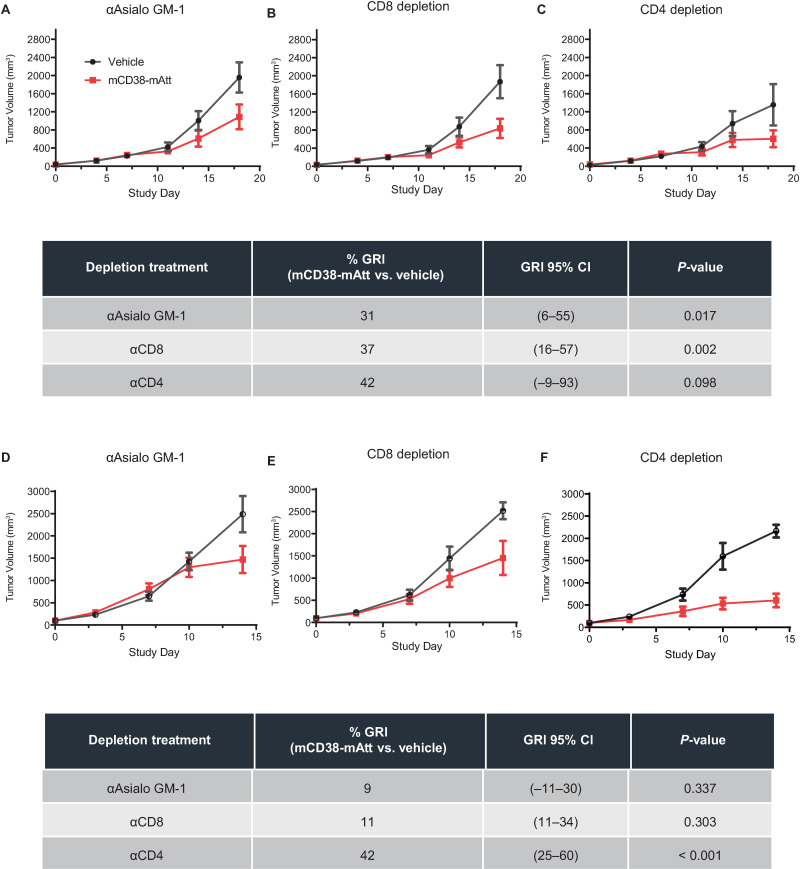
Depletion of T cells or NK cells negatively impacts the anti-tumor efficacy of mCD38-mAtt. Immunocompetent BALB/c mice were inoculated with **A–C**, A20, or **D–F**, CT26 cells and when tumors reached an average volume of ~ 100 mm^3^, mice were randomized into treatment groups and administered αAsialo GM-1- **(A, D)**, αCD8- **(B, E)**, or αCD4- (C, F) depleting antibodies to deplete specific immune cell subtypes, starting 1 day prior to drug treatment (day -1), and continuing weekly thereafter through the duration of the experiment. On day 0, mice were treated with a single dose of vehicle (PBS, 200 µ L final volume) or 10 mg/kg mCD38-mAtt and tumor volume was measured twice weekly until humane endpoint for vehicle control groups was reached.

To elucidate the relative contribution of various immune cell subsets to the anti-tumor activity demonstrated following mCD38-mAtt treatment, NK cells, CD8 T cells, or CD4 T cells were selectively depleted in A20 or CT26 tumor-bearing mice using depleting αAsialo GM-1 (for NK cell depletion), αCD8, or αCD4 antibodies, respectively, prior to treatment with mCD38-mAtt (Fig 5). Specific depletion of immune cell subsets was confirmed by flow cytometry in peripheral blood (S5 Fig). In mice bearing A20 tumors, there was evidence of anti-tumor activity despite depletion of NK cells, CD8 T cells, or CD4 T cells (growth rate inhibition (GRI) of 31% (P = 0.017), 37% (P = 0.002), or 42% (P = 0.098), respectively, versus their respective vehicle control; Fig 5A–C). Similarly, in mice bearing CT26 tumors, independent depletion of NK cells, CD8 T cells or CD4 T cells did not completely abrogate anti-tumor efficacy (9% (P > 0.337), 11% (P = 0.303), 42% (P < 0.001) GRI vs. vehicle control in CD4 depleted mice (Fig 5F)).

### Administration of mCD38-mAtt enhances the expansion and activation of CD8 T cells

Having demonstrated the contribution of different immune cells in mediating the anti-tumor activity of mCD38-mAtt, additional immunophenotyping was pursued across different immunocompetent tumor murine systems. Initial immunophenotyping studies focused on the J558 murine tumor model, which is insensitive to mIFNα *in vitro* yet demonstrates modest *in vivo* sensitivity to mCD38-mAtt treatment [19]. Leveraging this model, immunoprofiling analyses post treatment with mCD38-mAtt indicated increasing prevalence and activation of NK cells in peripheral blood and increased intra-tumoral CD8:Treg and CD8:CD4 T-cell ratios compared to treatment with anti-mCD38 antibody or murine IFNα alone [19].

**Fig 6:**
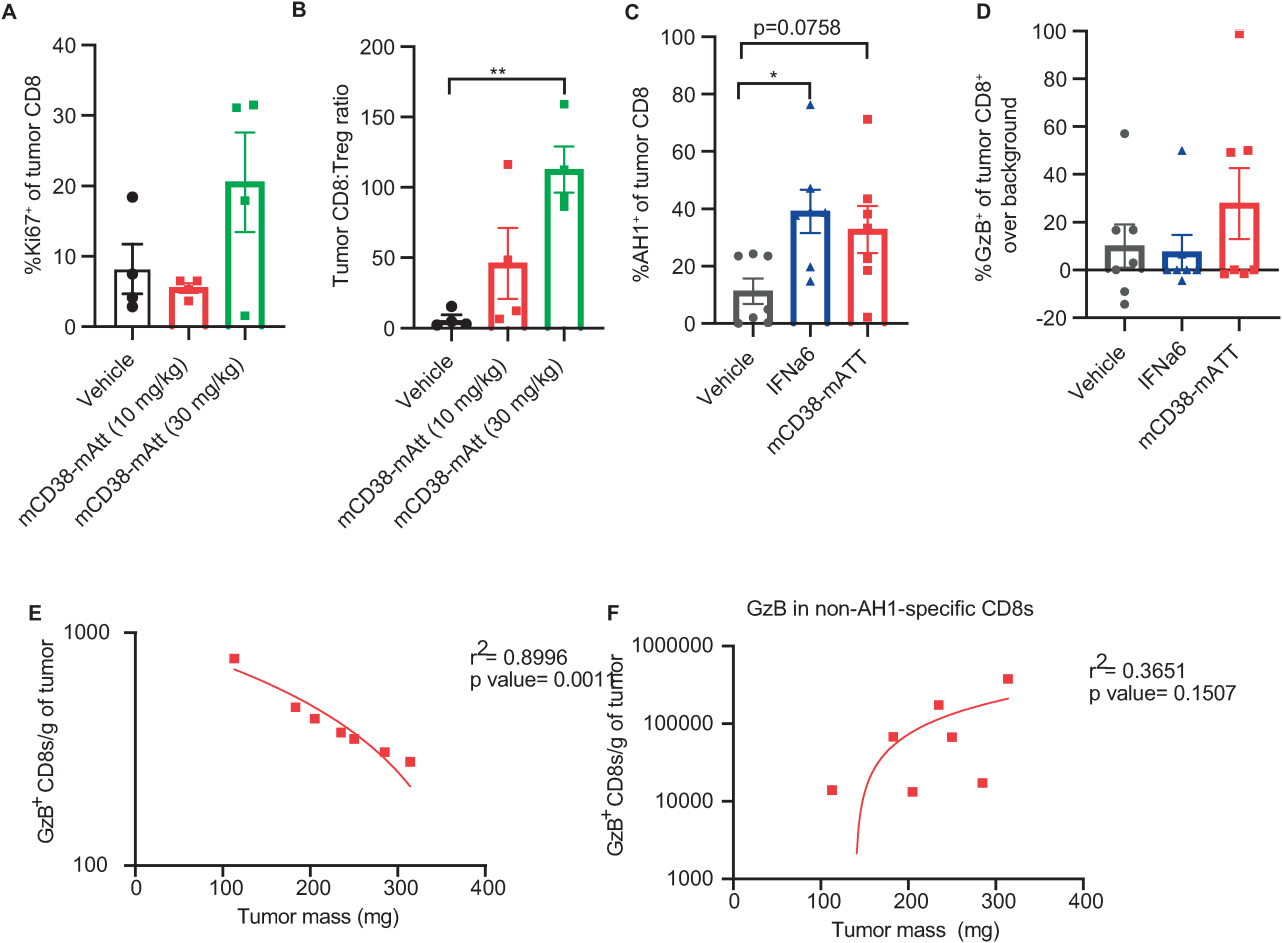
Treatment with mCD38-mAtt induces the proliferation and activation of CD8 T cells. Immunocompetent BALB/c mice were inoculated with CT26 tumor cells, and once tumors reached an average volume of ~ 300 mm^3^, mice were administered vehicle or mCD38-mAtt, at 10 or 30 mg/kg. Six days post treatment initiation, mice were euthanized, and tumors were collected. Proliferation of CD8 T cells was measured by Ki67 staining **A,** expressed as a percentage of total tumor CD8 T cells. **B,** The ratio of CD8:Tregs within tumors was also assessed. **C–E,** Immunocompetent BALB/c mice were inoculated with CT26 tumor cells, and once tumors reached an average volume of ~ 300 mm^3^, mice were administered vehicle, 2 mg/kg mIFNα, or 10 mg/kg mCD38-mAtt, and tumors were harvested 7 days post treatment. **C,** Tumor antigen AH1-specific CD8 T cells were measured by dextramer staining and are expressed as a percentage of total tumor CD8^+^ T cells. **D,** Dissociated tumor cells were stimulated *ex vivo* with or without AH1 peptide. The frequency of AH1-specific cells producing granzyme B was assessed by subtracting the background (percentage of granzyme B^+^ cells without AH1 restimulation) from AH1-stimulated cells. Correlation between the number of granzyme B^+^ **E,** AH1-specific, or **F,** non-specific CD8 T cells with tumor mass at the time of take down. Linear regression was used to assess goodness of fit. GzB, granzyme B; mIFNα, murine interferon alpha.

Building on these initial findings, potential changes in the intra-tumoral presence, phenotype, and functionality of different immune cell populations post-mCD38-mAtt treatment were evaluated. In these studies, leveraging the CT26 tumor model, mCD38-mAtt treatment enhanced CD8 T-cell proliferation (as assessed by percentage of positivity of the proliferation marker Ki67 on tumor CD8 T cells; Fig 6A) 7 days post treatment, which coincided with a concomitant increase in the tumor CD8 T cell to Treg ratio (Fig 6B), a positive prognostic indicator in many human cancers [20–22]. Tumor-infiltrating T cells can be tumor antigen-specific or bystander T cells that have trafficked to the tumor but have an irrelevant T cell receptor. Therefore, to determine whether the increased number of T cells in the tumors of mCD38-mAtt-treated mice were tumor antigen-specific, the presence of AH1-specific CD8 T cells was assessed. AH1 is a retroviral antigen expressed by most BALB/c tumors, and AH1-specific CD8 T cells can contribute to an anti-tumor immune response [23,24]. Compared with vehicle control, treatment with mCD38-mAtt increased the percentage of CD8 T cells in the tumor that were specific for AH1 (Fig 6C). Percentage of these cells that expressed the cytotoxic mediator granzyme B trended higher but did not reach statistical significance (Fig 6D). Notably, within the group of mice that were treated with mCD38-mAtt, the number of granzyme B-expressing, AH1-specific CD8 T cells inversely correlated with tumor weight (Fig 6E), whereas there was no significant relationship between the number of polyclonal, non-AH1-specific CD8 T cells expressing granzyme B with tumor weight (Fig 6F). Taken together, these data suggest that mCD38-mAtt expands and activates a population of tumor antigen-specific CD8 T cells that may play a role in tumor clearance.

## Discussion

In this study, we sought to delineate the effects of a CD38-targeted attenuated IFNα immunocytokine (CD38-Attenukine^TM^) on tumor cells and/or immune cells, through leveraging different immunocompromised and immunocompetent mouse models. In immunocompromised, CD38-expressing tumor model settings, robust anti-tumor activity of hCD38-hAtt was demonstrated. The depth of response somewhat aligned, but was not directly correlated, with tumor CD38 expression levels in vitro (Fig 1), though we do acknowledge that levels of target expression on cell lines may not necessarily remain consistent when evaluated in *in vitro* and *in vivo* settings. Follow-on studies in an immunocompetent mouse model bearing 38C13 murine tumors engineered to overexpress human CD38 demonstrated that the anti-tumor activity of a hCD38-mAtt surrogate was due in part to increased intra-tumoral presence and activation of NK cells, CD4 T cells and CD8 T cells, based on immune cell depletion and immunophenotyping analyses (Figs 2 and 3). As the hCD38 portion of this surrogate can only bind to the human CD38 expressed on the 38C13 tumors, these data suggest that hCD38-mAtt acts directly on the tumor cells but may also indirectly activate immune cell populations. This could potentially be through increased antigen presentation or stress ligand expression as a result of increased immunoproteasome subunit expression [25], immunogenic cell death [26,27], production of pro-inflammatory cytokines [28], or changes in expression of stimulatory ligands [29].

Many hematological malignancies, such as multiple myeloma, T cell acute lymphoblastic leukemia (ALL), peripheral T cell lymphoma [30], and B cell ALL, express CD38 [31–33]; however, most solid tumors lack tumor-intrinsic expression of CD38, with the exception of certain prostate tumors [34] and lung tumors, where upregulation of CD38 has been associated with resistance to programmed cell death protein/ligand-1 therapy [35,36]. In such settings, the potential anti-tumor benefit of a CD38-targeted Attenukine^TM^ would rely on its immune-directed effects alone.

To evaluate this specific question, the anti-tumor activity of a fully murine CD38-Attenukine™ surrogate, namely mCD38-mAtt, was evaluated in syngeneic mouse models bearing different murine tumors that do not express CD38. In these studies, there was clear evidence of significant anti-tumor activity following a single administration of 10 mg/kg mCD38-mAtt across four of the five models tested (Fig 4). Follow-on studies leveraging the A20 and CT26 models suggest that NK cells contribute to anti-tumor activity, as selective depletion of NK cells reduced the tumor growth inhibition of mCD38-mAtt treatment (comparing delta in anti-tumor activity of mCD38-mATT in non-depleted (Fig 4A) versus NK depleted (Fig 5A) tumors, across two independent experiments). These data, in addition to previously published work [19], clearly highlight the innate immune activation potential of a CD38-directed Attenukine^TM^ and are consistent with previous observations of the effects of IFNα on NK cells [37].

In addition to effects on innate immune cells, treatment with hCD38-mAtt or mCD38-mAtt stimulated adaptive immune cells. Leveraging the 38C13-hCD38-expressing immunocompetent mouse model, treatment with hCD38-mAtt was negatively impacted when CD4 and CD8 T cells were selectively depleted, albeit to a lesser degree than NK cell depletion (Fig 2). In addition, treatment with hCD38-mAtt resulted in an increased number of NK cells, as well as CD4 T cells and CD8 T cells in the tumor, compared with controls (Fig 3A–C). In this model, hCD38 is only expressed on tumor cells; thus, the immune modulatory effects of hCD38-mAtt are likely indirect, as described above, which can facilitate the activation and anti-tumor function of NK cells and T cells. The tumor-infiltrating CD8 T cells acquired an effector phenotype, evidenced by increased expression of the cytotoxic mediator granzyme B and decreased expression of the anti-inflammatory and pro-tumor cytokine TGFβ1. In CT26-bearing mouse studies, elevated Ki67 expression suggests that these T cells proliferated in situ. It has previously been observed that IFNα signaling can have antiproliferative effects on T cells prior to receiving T cell receptor (TCR) stimulation, but that proliferation is enhanced if IFNAR signaling occurs after TCR signaling, driven by the differential activity of STAT1 [38]. Thus, our observation of increased proliferation suggests that these tumor-infiltrating CD8 T cells are being activated by the tumor, rather than being nonspecific bystanders. Furthermore, CT26 tumors express the AH1 antigen derived from an endogenous retrovirus [39]. In *ex vivo* analyses, increased numbers of AH1-specific T cells were observed relative to intra-tumoral CD8 T cells, as well as elevated granzyme B expression in these tumor antigen-specific cells, which correlated with a reduction in tumor mass. Finally, an increase in the CD8:Treg ratio upon mCD38-mAtt treatment was observed, a metric which has been associated with a favorable clinical prognosis across multiple tumor types (reviewed in Elkoshi *et al*. [40]).

In summary, our data indicate that a CD38-directed Attenukine^TM^ can deliver the desired tumor-directed and immune-directed impacts of IFNαtherapy without inducing tolerability issues. Through use of different *in vivo* systems and CD38-directed Attenukine^TM^ surrogates, a deeper understanding of the potential mechanistic effects of a CD38-directed Attenukine^TM^ was realized, with evidence of tumor growth inhibition, demonstrated through direct effects of IFNα on tumor cells as well as by modulating an anti-tumor immune response, in the absence of any adverse effects based on gross observation and body weight change alone. Taken together, these data point to a dual mechanism of action for a CD38-targeted Attenukine^TM^, involving both immune and tumor-directed activity, and highlight the potential benefit of a CD38-targeted attenuated IFNα therapy.

## Supporting information

Fig S1 Confirmed over-expression of human CD38 on engineered 38C13-hCD38 murine tumor cell line. Expression of human CD38 on 38C13-hCD38 and parental 38C13 cells, compared to isotype control.
(TIF)

Fig S2 Gating strategy for different immune cell populations. Sequential flow cytometry plots and associated gating strategies are provided. After excluding doublet and dead cells, the indicated gating strategies were used. A, NK cell gating. B, T cell gating. Gates were set based on fluorescence minus one control. TNF, tumor necrosis factor.
(TIF)

Fig S3 *In vitro* sensitivity to mIFNα of engineered 38C13-hCD38 murine tumor cell line. Differential inhibition of proliferation of 38C13-hCD38 cells by mIFNα or hCD38-mAtt across a range of equimolar concentrations of IFNα. hCD38-mAtt, mIFNα,recombinant murine IFNα. Data represent mean of 3 independent replicate samples per treatment concentration, with error bars indicating SD.
(TIF)

Fig S4 Murine tumor cell line *in vitro* sensitivity to mIFNα. Murine tumor cell lines A, A20, B, B16F10, C, MC38, D, CT26, and E, PANC02 were propagated with mIFNα at concentrations ranging from 0.1 to 100,000 IU/mL for 72 hours. Proliferation was measured by CellTiter-Glo assay. The percent inhibition of cell proliferation for each cell line following 72-hour treatment with IFNα *in vitro* compared to untreated control (as a measure of intrinsic sensitivity to IFNα treatment). mIFNα,recombinant murine IFNα.
(TIF)

Fig S5 Confirmation of immune cell depletion in peripheral blood following treatment with specific immune cell depleting antibodies. One day prior to control or Attenukine molecule treatment, tumor bearing mice were dosed intraperitoneally with immune cell-specific depleting antibodies (i.e., 150 μg αCD8, 300 μg αCD4, 50 μl αAsialo GM-1 antibodies). Levels of A, CD8 T cells, B, CD4 T cells, or C, Natural killer cells in peripheral blood were assessed by flow cytometry 4 days after depleting antibody administration to confirm depletion of immune cell subsets.
(TIF)
