# Supplementary figures and images for: CD38-targeted attenuated interferon alpha immunocytokine activates both innate and adaptive immune cells to drive anti-tumor activity
Source: PLoS One. 2025 May 2;20(5):e0321622. doi: 10.1371/journal.pone.0321622 (PMC12047799; doi:10.1371/journal.pone.0321622)

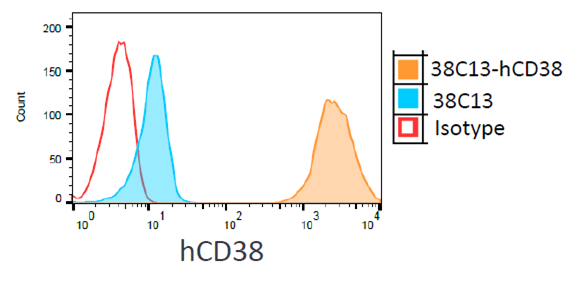

Supplement: Fig S1 — Expression of human CD38 on 38C13-hCD38 and parental 38C13 cells, compared to isotype control. (TIF) [file pone.0321622.s001.tif]

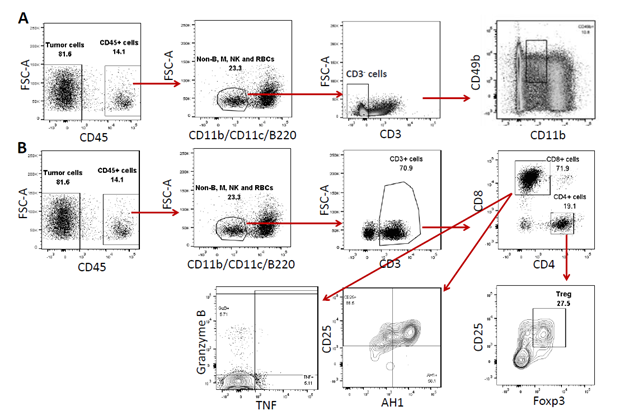

Supplement: Fig S2 — Sequential flow cytometry plots and associated gating strategies are provided. After excluding doublet and dead cells, the indicated gating strategies were used. A, NK cell gating. B, T cell gating. Gates were set based on fluorescence minus one control. TNF, tumor necrosis factor. (TIF) [file pone.0321622.s002.tif]

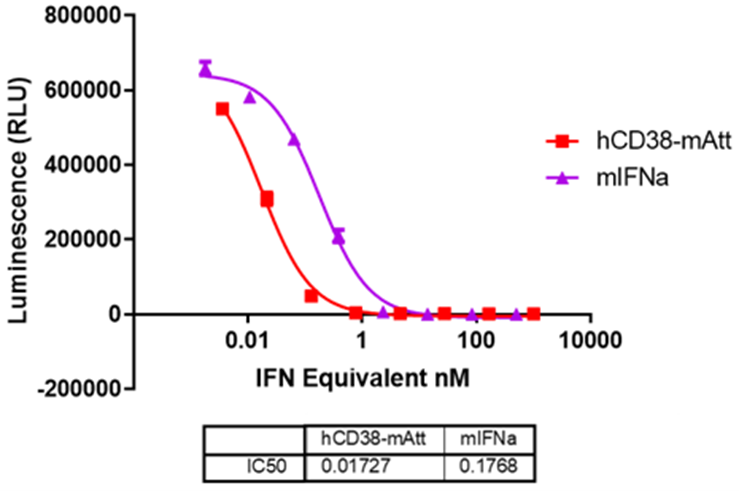

Supplement: Fig S3 — Differential inhibition of proliferation of 38C13-hCD38 cells by mIFNα or hCD38-mAtt across a range of equimolar concentrations of IFNα. hCD38-mAtt, mIFNα,recombinant murine IFNα. Data represent mean of 3 independent replicate samples per treatment concentration, with error bars indicating SD. (TIF) [file pone.0321622.s003.tif]

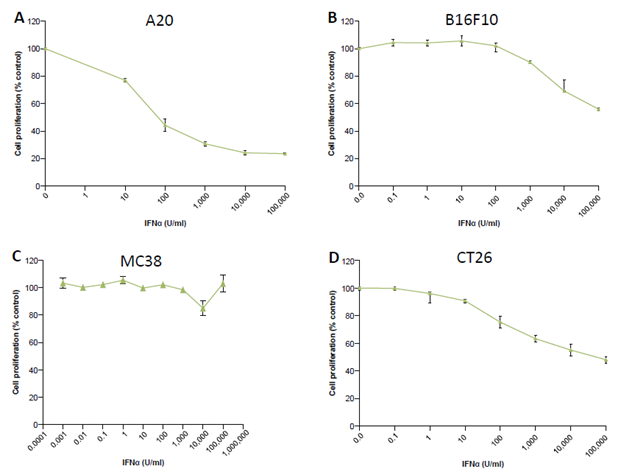

Supplement: Fig S4 — Murine tumor cell lines A, A20, B, B16F10, C, MC38, D, CT26, and E, PANC02 were propagated with mIFNα at concentrations ranging from 0.1 to 100,000 IU/mL for 72 hours. Proliferation was measured by CellTiter-Glo assay. The percent inhibition of cell proliferation for each cell line following 72-hour treatment with IFNα in vitro compared to untreated control (as a measure of intrinsic sensitivity to IFNα treatment). mIFNα,recombinant murine IFNα. (TIF) [file pone.0321622.s004.tif]

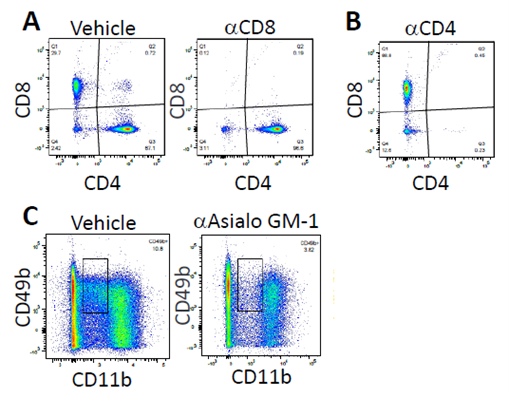

Supplement: Fig S5 — One day prior to control or Attenukine molecule treatment, tumor bearing mice were dosed intraperitoneally with immune cell-specific depleting antibodies (i.e., 150 μg αCD8, 300 μg αCD4, 50 μl αAsialo GM-1 antibodies). Levels of A, CD8 T cells, B, CD4 T cells, or C, Natural killer cells in peripheral blood were assessed by flow cytometry 4 days after depleting antibody administration to confirm depletion of immune cell subsets. (TIF) [file pone.0321622.s005.tif]
